# Morphology-Dependent Room-Temperature Ferromagnetism in Undoped ZnO Nanostructures

**DOI:** 10.3390/nano11123199

**Published:** 2021-11-25

**Authors:** Hongtao Ren, Gang Xiang

**Affiliations:** 1 School of Materials Science and Engineering, Liaocheng University, Hunan Road No. 1, Liaocheng 252000, China; 2 College of Physics, Sichuan University, Wangjiang Road No. 29, Chengdu 610064, China

**Keywords:** dimensional engineering, substrate engineering, thickness-dependence

## Abstract

Since Dietl et al. predicted that Co-doped ZnO may show room-temperature ferromagnetism (RTFM) in 2000, researchers have focused on the investigation of ferromagnetic ZnO doped with various transition metals. However, after decades of exploration, it has been found that undoped ZnO nanostructures can also show RTFM, which in general is dependent on ZnO morphologies. Here, we will give an overall review on undoped ZnO nanomaterials with RTFM. The advanced strategies to achieve multidimensional (quasi-0D, 1D, 2D, and 3D) ferromagnetic ZnO nanostructures and the mechanisms behind RTFM are systematically presented. We have successfully prepared ferromagnetic nanostructures, including thin films, horizontal arrays and vertical arrays. The existing challenges, including open questions about quantum-bound ZnO nanostructures, are then discussed.

## 1. Introduction

In recent years, due to their potential applications in spintronic devices, diluted magnetic semiconductors have attracted the attention of researchers [1]. Many research groups have attempted to look for compounds with room-temperature ferromagnetism (RTFM) since 2000, which can be obtained with doping transition metal elements into metal oxides including ZnO [2,3,4,5,6], TiO_2_ [7,8,9,10], SnO_2_ [11,12,13,14,15,16], In_2_O_3_ [17,18] and HfO_2_ [10,19,20,21]. Since it was predicted based on the Zener model that Co-doped ZnO could exhibit RTFM [22], researchers have gradually focused on transition metal-doped ZnO systems. As an overview of the many publications on doped ZnO with RTFM, some review articles [4,6,23,24,25,26,27,28,29,30,31,32,33] are presented in Table 1.

**Figure 1:**
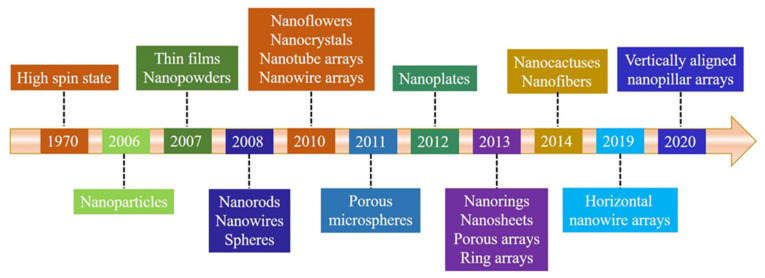
Timeline showing key developments of ZnO nanostructures with RTFM.

In fact, undoped ZnO can also exhibit ferromagnetic ordering. The spin state related with Zn vacancy (V_Zn_) was identified first by its electron spin resonance (ESR) spectrum in electron-irradiated ZnO single crystals [34]. It is worthwhile noting that recent experimental results have shown that nano-engineering can also induce ferromagnetic behavior in non-ferromagnetic bulk materials [35,36,37]. The magnetic ordering obtained by the minimum number of vacancies was not successfully detected at that time. Amazingly, it was found later that the miniature of the bulk materials in nanoscale, such as nanoparticles (NPs), could exhibit novel magnetic behaviors in undoped ZnO. In 2006, RTFM (*M_s_*, ~0.0005 emu/g) was observed in sol-gel ZnO NPs [38], which could be attributed to oxygen vacancies (V_O_). Subsequently, Banerjee et al. [39]. found that thermal annealing could induce RTFM in ZnO white powder, owing to the formation of V_O_ clusters during the thermal treatment. Then, RTFM with a saturation magnetization up to 35.65 emu/g was also observed in undoped ZnO thin films (TFs) by pulsed laser deposition (PLD) and V_Zn_ located at the surface or the interface of the samples could be the source of RTFM [40]. More importantly, various ZnO nanostructures with RTFM were obtained by various approaches, such as ionic layer epitaxy [41], polymer-assisted deposition (PAD) [42,43,44], electrochemical deposition method [45], sol-gel [36,46,47], ball milling (BM) [48,49,50], and PLD [51], etc. Here, we will give an overview of the timeline of undoped ZnO nanostructures with RTFM (Figure 1).

## 2. Dimension Design

ZnO nanostructures can be divided into the following categories according to dimensions, such as: quasi-0D materials (quantum dots (QDs), and nanoparticles (NPs), 1D materials (nanorods (NRs), nanowires (NWs), nanostructured arrays, capped nanowires/nanotubes arrays, and other nanostructures), 2D materials (nanosheets, nanoplates, and thin films (TFs)), and 3D materials (single crystals, and porous microspheres).

### 2.1. Zero- and Quasi Zero-Dimensional Materials

**Figure 2:**
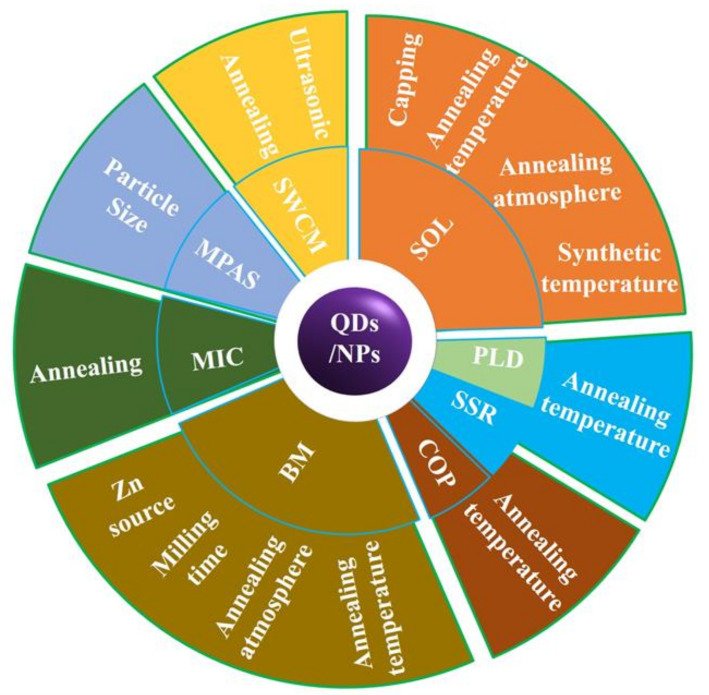
Overview of currently available preparation strategies for fabricating ZnO QDs/NPs with RTFM. Sol-gel (SOL); Pulsed laser deposition (PLD); Solid-state reactions (SSR); Coprecipitation (COP); Ball milling (BM); Miceller method (MIC); Microwave plasma assisted spray (MPAS); Surfactant-free wet chemical method (SWCM).

**Figure 3:**
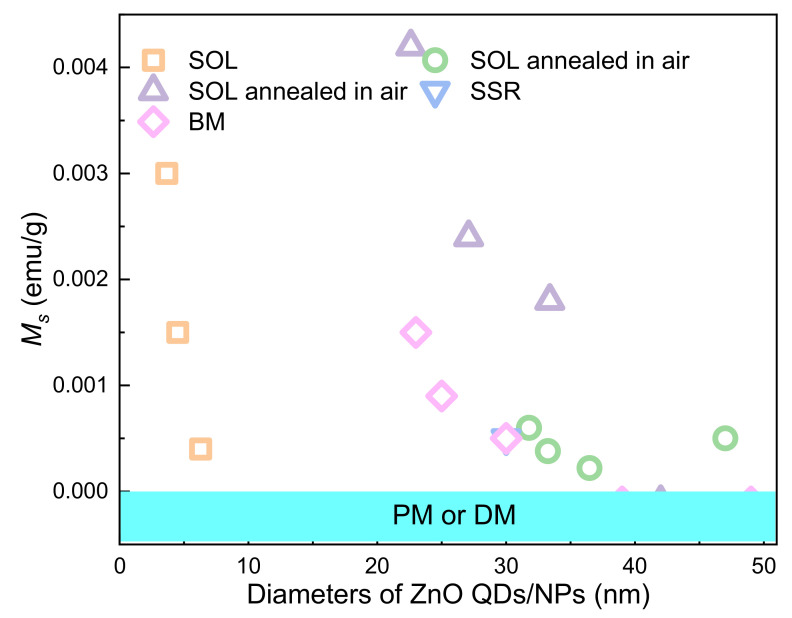
The size-dependent RTFM in ZnO QDs/NPs.

Qusai-0D materials, such as Quantum dots (QDs), are crystalline particles with sizes less than 100 nm. ZnO QDs with RTFM (Figure 2) are commonly deposited by sol-gel (SOL) [36,47,52,53,54], solid-state reactions (SSR) [38], ball milling (BM) [49,55,56,57,58,59,60,61] pulsed laser deposition (PLD) [51], and coprecipitation (COP) [62,63] and so on. Previous studies have shown that RTFM of QDs can be controlled by synthetic temperature [52], milling time, annealing temperature or atmosphere [53,54], Zn source and capping [36,47]. As another kind of quasi zero dimensional material, nanoparticles (NPs) are about hundreds of nanometers in size. ZnO NPs with RTFM have been synthesized by BM [49,59], microwave plasma assisted spray (MPAS) [64], the miceller method (MIC) [39], COP [62] and the surfactant-free wet chemical method (SWCM) [65] (Figure 2). It has also been found that RTFM can be controlled by annealing parameters [39,59,62,65], particle size [64] and milling time [49]. The size-dependent RTFM in ZnO QDs/NPs (Figure 3) can be observed [52]. *M_s_* is increased gradually by decreasing the diameters of ZnO NPs, and then RTFM will transform into paramagnetism (PM) or diamagnetism (DM).

### 2.2. One-Dimensional Materials

#### 2.2.1. Nanowires/Nanorods

**Figure 4:**
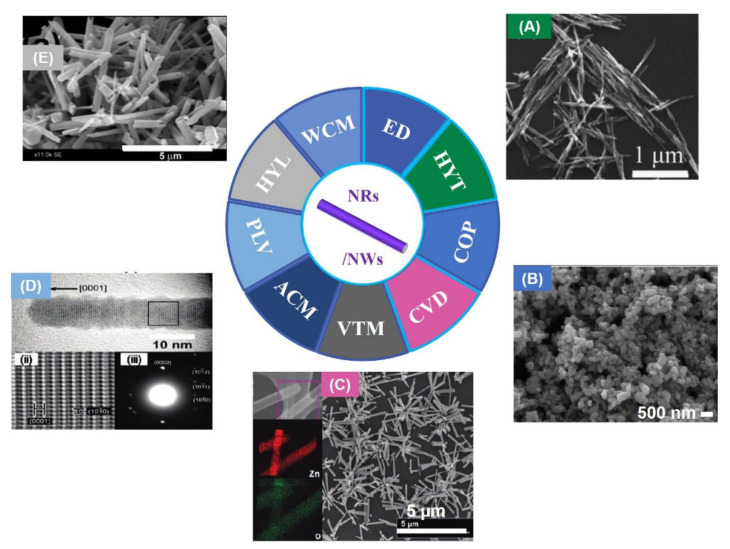
ZnO NRs/NWs. (**A**) ZnO NRs obtained by hydrothermal method (HYT) (Reproduced with permission from [67]. Copyright 2008, American Institute of Physics). (**B**) ZnO NRs prepared by coprecipitation method (COP) (Reproduced with permission from [68]. Copyright 2009, Elsevier). (**C**) SEM images of ZnO NWs (Reproduced with permission from [69]. Copyright 2010, American Chemical Society). (**D**) HRTEM image of ZnO NRs (Reproduced with permission from [69]. Copyright 2010, American Chemical Society). (**E**) ZnO NRs synthesised by wet chemical method (WCM) (Reproduced with permission from [72]. Copyright 2018, Elsevier). Electro-deposition (ED); Chemical vapor deposition (CVD); Pulsed laser vaporization (PLV); Aqueous chemical method (ACM); Hydrolysis (HYL).

RTFM has been found in ZnO NWs [66] prepared by electro-deposition (ED). Due to the incomplete oxidation of zinc NWs, zinc clusters are formed in ZnO NWs, which induce room temperature ferromagnetism. Subsequently, ZnO NRs exhibiting RTFM have been obtained by hydrothermal method (HYT) [67] with Zn(Ac)_2_·2H_2_O and NaOH (Figure 4A). When the diameters of the NRs decreases, the *M_s_* of the as-grown sample increases. Zn_i_ at the surface of ZnO NRs may be one of the reasons for RTFM.

In addition, V_Zn_ can also be produced in ZnO NRs with RFTM (Figure 4B), which were synthesized by COP [68]. The as-prepared NWs [69] (Figure 4C) show an obvious RTFM (*M_s_*, ~0.007 emu/g). However, highly crystalline ZnO NWs [69] (Figure 4) prepared by the pulsed laser vaporization (PLV) process show no RTFM. X-ray fine structure spectroscopy study confirms that V_Zn_ can cause RTFM in ZnO NRs.

Furthermore, ZnO NRs have been obtained in aqueous conditions [70]. As-prepared NRs show RTFM behavior. Strangely, when the size of the NRs decrease, the magnetization of ZnO NRs increases. Singly charged oxygen vacancies (V_O_^+^) localized on the sample is the origin of RTFM. Single-crystalline ZnO NWs with RTFM (*M_s_*, ~0.001 emu/g) (Figure 4D,E) have been obtained by the vapor transport method (VTM) [71]. RTFM related with V_O_ can be modulated by selecting different catalysts and changing the growth temperature. Jana et al. [72] has reported ZnO NRs (*M_s_*, ~0.059 emu/g) using hydrolysis of zinc acetate. Moreover, polycrystalline ZnO NRs with RTFM have also been prepared by a wet chemical method (WCM) [73]. RTFM in ZnO NRs (Figure 4) is attributed to V_O_^+^.

#### 2.2.2. Nanostructure Arrays

**Figure 5:**
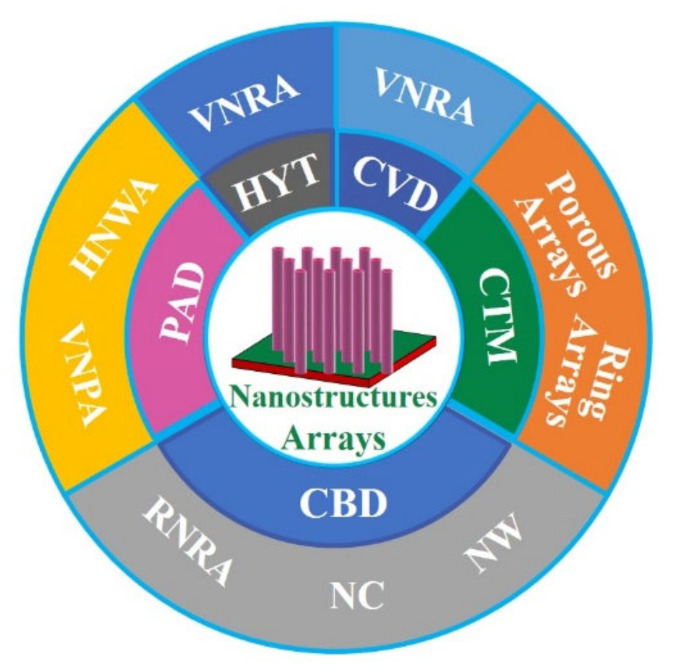
ZnO nanostructure arrays.

ZnO nanostructure arrays (Figure 5) have been fabricated by various approaches, such as HYT [74], colloidal template method (CTM) [75], chemical vapor deposition (CVD) [76], chemical bath deposition (CBD) [76,77], and polymer-assisted deposition (PAD) [42,43,44]. ZnO NWs are usually synthesized by a seed method. Firstly, ZnO TFs are prepared by magnetron sputtering and used as seed layer, then ZnO NRs arrays are obtained by HYT [74]. The defect states are controlled by annealing in O_2_ and H_2_ atmosphere at 500 °C, respectively. It has been found that V_O_ is closely related to RTFM by PL spectrum and vibrating sample magnetometer (VSM). Furthermore, vertically oriented NRs arrays (VNRA) and randomly oriented NRs arrays (RNRA) have been synthesized by CVD and CBD [76]. These results show that RTFM is originated from the V_O_^+^.

**Figure 6:**
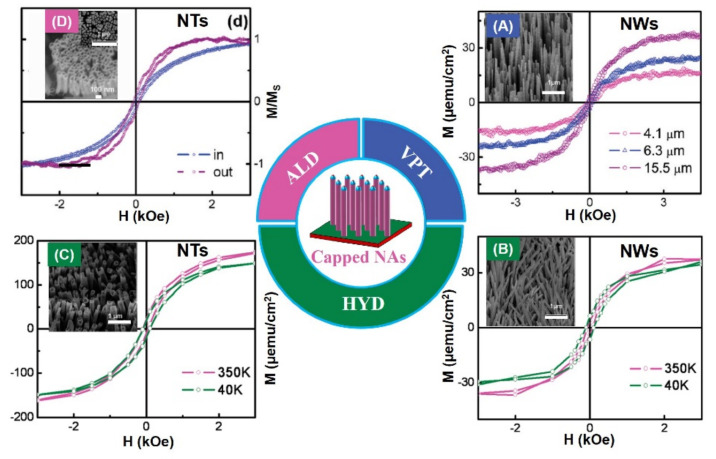
RTFM of ZnO nanowire (NWs)/nanotube arrays (NTs) is modulated by capping with thiol. (**A**) M–H curves for ZnO NWs of different heights. (**B**) M–H curves for ZnO NW arrays of diameters ~150 nm. (**C**) M–H curves for ZnO NT arrays. (**D**) NT arrays (Reproduced with permission from [78]. Copyright 2009, Elsevier).

Interestingly, ZnO porous arrays (*M_s_*, ~5.7 emu/g) and ring arrays with RTFM (*M_s_*, ~0.25 emu/g) have been fabricated by CTM [75] (Figure 5). When the grain size decreases to ~3 nm, the *M_s_* can increase to ~5.7 emu/g. The results show that grain size and V_O_ concentration can affect the RTFM. The presence of the defects in ZnO nanocactuses (*M_s_*, ~3.0 emu/cm^3^) than in the ZnO NWs (*M_s_*, ~2.3 emu/cm^3^) was observed [77] in Figure 5. The low temperature method can generate more defects, and then induce the higher *M_s_* in nanocactuses. ZnO HNWA [43] and VNPA [44] have also been obtained by PAD in our lab. Our results show that more V_Zn_ can potentiate RTFM [43,44]. Thiol-capped ZnO NWs/NTs arrays can present RTFM [78]. The M-H loops of the thiol-capped samples reveal the height-dependent and morphology-dependent RTFM (Figure 6). Combing magnetic measurements and calculations show that the origin of ferromagnetism is mainly attributed to spin-polarized 3p electrons in S sites and, therefore, has a strong correlation with Zn–S bond anisotropy.

**Figure 7:**
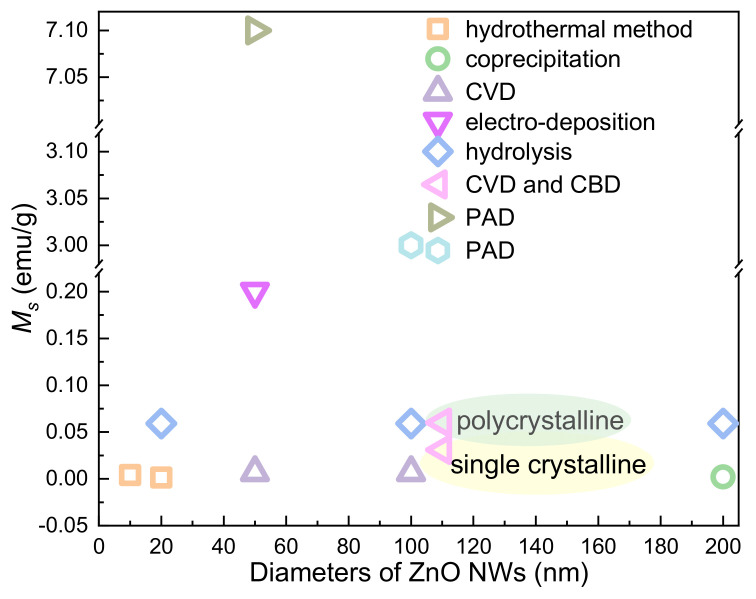
The size-dependent RTFM in ZnO NWs.

In the nanowire system, the change of diameter has no obvious effect on RTFM (Figure 7). However, the height-dependent RTFM has been observed in capped ZnO NWs (Figure 6A).

#### 2.2.3. Other Nanostructures

**Figure 8:**
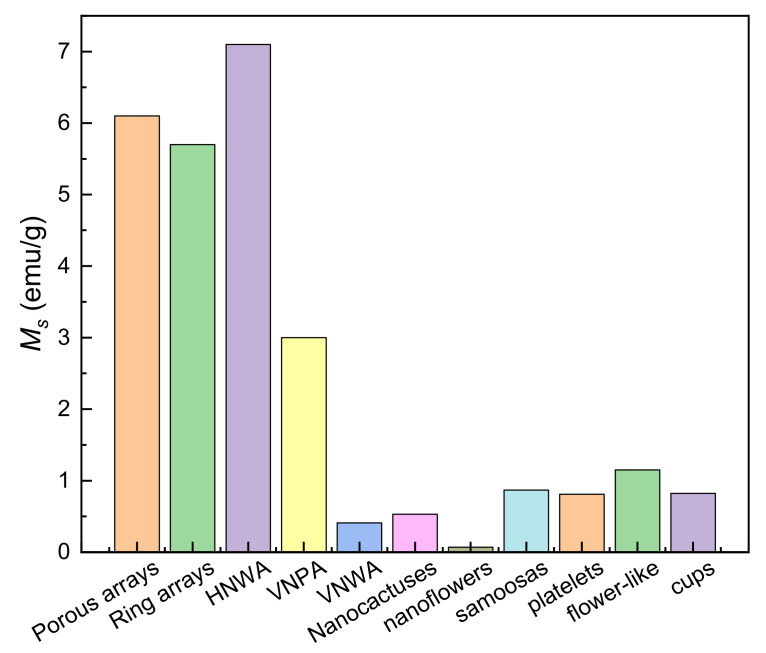
Saturation magnetizations (*M_s_*) of various ZnO nanostructures. Data from refs. [43,44,75,77,79,81].

ZnO nanoflowers with RTFM (*M_s_*, ~0.069 emu/g) have been obtained by HYT [79]. TEM images show that this flower sample is composed of closely packed NRs. XPS indicates that isolated vacancy clusters is closely related to the RTFM. In addition, microdiscs and porous nanoassembly of ZnO have been synthesized by soft-chemical approaches [80]. Furthermore, various types of ZnO nanostructures have been obtained by a microwave assisted HYT [81]. Spectrum measurements show that the defects may mediate RTFM [80,81]. The morphology-dependent RTFM is shown in Figure 8. ZnO HNWA has the highest *M_s_* is ~7.1 emu/g.

### 2.3. Two-Dimensional Materials

#### 2.3.1. Nanosheets/Nanoplates

**Figure 9:**
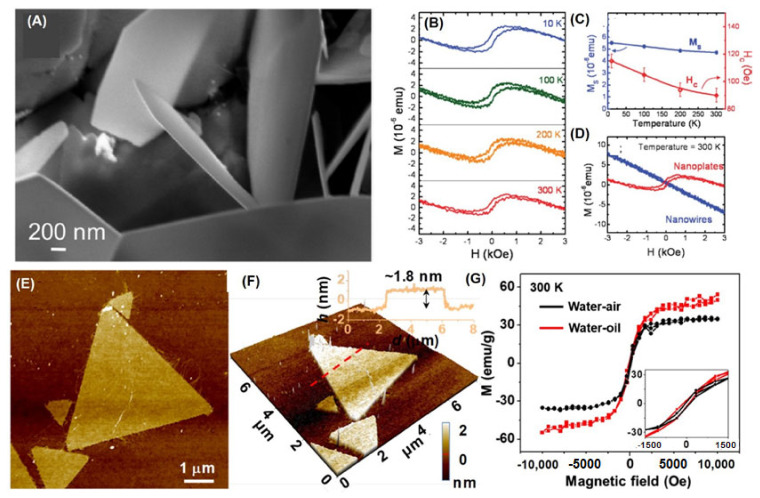
(**A**) ZnO nanoplates synthesized by HYT. (**B**) M–H curves for ZnO nanoplates. (**C**) The corresponding *M_s_* and coercivities *H_c_* are plotted in (**A**). (**D**) M–H curves for ZnO nanowires and nanoplates (Reproduced with permission from [82]. Copyright 2012, American Chemical Society). (**E**,**F**) AFM images of two-dimensional (2D) ZnO nanosheets by ILE. (**G**) M-H curves for 2D ZnO nanosheets (Reproduced with permission from [41]. Copyright 2019, American Chemical Society).

Two-dimensional (2D) nanosheets/nanoplates have been synthesized by HYT [45,82,83] and ionic layer epitaxy (ILE) [41] in Figure 9. The average thickness of ZnO Nanoplates is estimated to be ~20 nm by the SEM images and XRD peak. Interestingly, it is only when the thickness is reduced to 5–8 nm, the samples exhibit RTFM. Interestingly, first principles calculation results show that when the thickness of the NSs decreases to a certain range, the magnetic moment will be generated; however, when the size of the NWs decreases along the a-axis and b-axis, the magnetic moment will not be generated. The distorted bands may be responsible for the RTFM in ZnO nanoplates.

ZnO single-crystalline nanosheets with RTFM have been obtained [45]. TEM images present that ZnO sheets and dodecyl sulfate bilayers compose the lamellar structure. The results show the cluster of spin-polarized defects induce RTFM in single-crystalline ZnO nanosheets.

Two-dimensional ZnO nanosheets with high V_Zn_ concentration have been grown by ILE [41] (Figure 9E,G). The nanosheets annealed in Ar at 400 °C for 1h show *M_s_* of 50.9 emu/g in Figure 9G. Significantly, DFT calculations and experimental results suggest that V_Zn_ could be associated with the RTFM.

**Figure 10:**
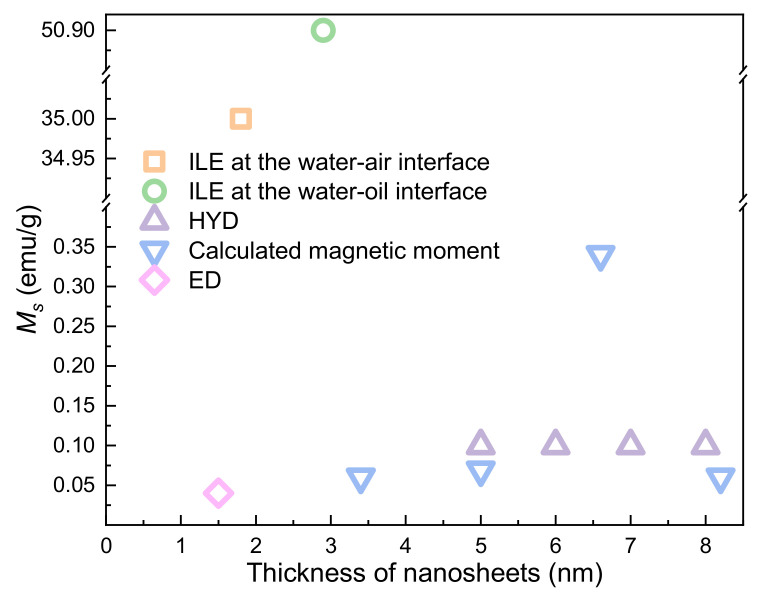
The thickness-dependent RTFM in ZnO nanosheets.

By contrast with 2D materials, ZnO nanosheets do not show obvious thickness dependence. However, ZnO nanosheets with saturation magnetization up to 50.9 emu/g have been obtained recently by ILE (Figure 10).

#### 2.3.2. Thin Films

In 2007, RTFM has been first observed in undoped ZnO TFs by PLD [40]. The results suggest that V_Zn_ can generate RTFM, where the ferromagnetism is related to the thickness of ZnO TFs. Soon after, calculations [84] not only showed that undoped ZnO TFs and NWs can be ferromagnetic but also indicated that V_Zn_ is responsible for RTFM in low-dimensional magnetic ZnO nanostructures. Similar to other nanostructures [38,40,67,80,81], V_Zn_ prefers to reside on ZnO NWs, resulting in stronger RTFM.

The thickness-dependent ferromagnetism in ZnO TFs has been investigated by Kapilashrami et al. [85]. With increasing the film thickness, the ferromagnetism first increases; when the film thickness exceeds 480 nm (*M_s_*, ~0.62 emu/g), the ferromagnetism then decreases; with increasing of film thickness, the ferromagnetism will transform into paramagnetism (PM) or even diamagnetism (DM). The defect is mainly responsible for the observed RTFM.

Grain boundaries can also mediate RTFM. ZnO TFs were also obtained by liquid ceramics method [86]. The HRTEM images show that ZnO grains is surrounded by the amorphous area. Furthermore, RTFM origins from the amorphous regions related the defects. By decreasing the grain size, the magnetic volume fractions will increase [87]. Tietze et al. [87]. built a magnetic shell model related to the grain boundaries, which clarified the origin of RTFM in ZnO TFs.

In the ZnO TFs system, V_Zn_ [40,84,88,89,90] can also induce the RTFM. The existence of V_Zn_ is often confirmed by photoluminescence and positron annihilation spectrum. Xing et al. [88] has found that the sol-gel derived samples contain more V_Zn_, compared with the MBE-ZnO TFs, which shows much stronger RTFM. Similarly, V_Zn_ is also the origin of RTFM in polycrystalline ZnO TFs prepared by PAD in our lab [89,90].

Additionally, undoped ZnO TFs [91,92] have been fabricated with CVD [93]. Interestingly, a nanosized structure can induce RTFM in ZnO TFs. Similar to ZnO NRs [71,76], V_O_ can generate RTFM in undoped ZnO TFs obtained by PED [94] and undoped ZnO NPs prepared by an electrospinning method [95]. Moreover, Zhang et al. [96] indicated that RFTM in pure ZnO TFs should be related to Zn_i_. The shallow donor caused by Zn_i_ defects might modify the electronic structure of ZnO TFs, leading to the RTFM.

**Figure 11:**
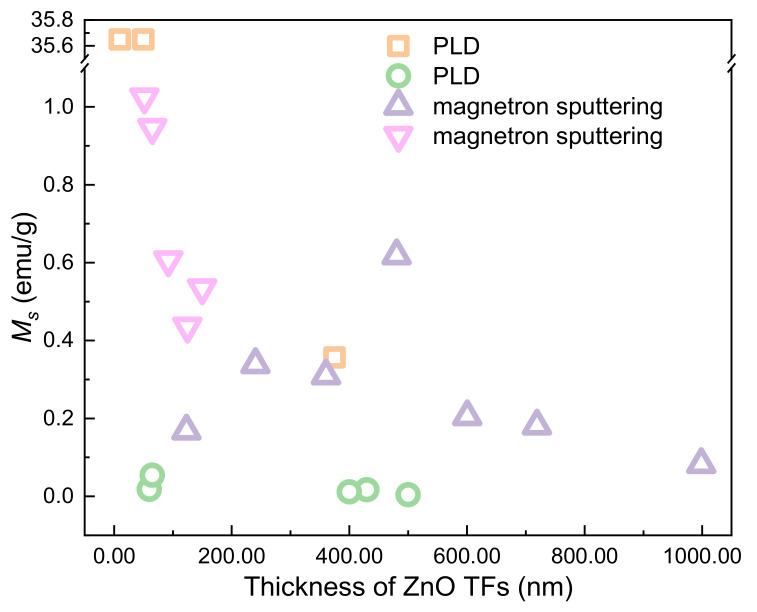
The thickness-dependent RTFM in ZnO TFs.

The thickness dependence of RTFM has been observed in films prepared by different experimental methods (Figure 11). The RTFM of ZnO TFs is affected by the defects on the surface of the sample or at the interface between the sample and the substrate.

### 2.4. Three-Dimensional Materials

In 1970, Galland et al. [34] irradiated ZnO single crystals with 3MeV electrons. ESR spectra showed that V_Zn_ can generate the high spin state. Furthermore, the intrinsic defect just like Zn_i_ and V_O_ may give rise to the RTFM in single crystalline ZnO [97].

Interestingly, the metal–insulator transition has been found in the ZnO supercells adsorbed with hydrogen atoms by first-principles calculations [98]. This phase transition can affect the magnetic transformation. Experimentally, ZnO single crystals have been obtained by HTY [99]. The hydrogen penetration depth is confirmed at 20 nm by stopping and range of ions in matter (SRIM) simulation. SQUID measurements indicate that the *M_s_* increases with the treatment time and the concentration of hydrogen. Subsequently, hydrogen atoms may trigger the RTFM in ZnO single crystals in Khalid et al. [100]. The *M_s_* is up to ~4 emu/g. Strangely, the vacancies defects and the interstitial Zn atoms cannot induce the RTFM in ZnO monocrystal [101].

HYT [102] has been developed to grow Zn_5_(OH)_8_Ac_2_·2H_2_O microspheres. In fact, the microspheres are grown from the nanosheets, which are curved and connected to each other. ZnO porous microspheres [103] have been obtained from Zn_5_(OH)_8_Ac_2_·2H_2_O microspheres annealing at 500 °C. The RTFM in pure ZnO microspheres origins from V_Zn_ and shallows donors.

## 3. Ferromagnetism of Undoped ZnO Nanostructures

### 3.1. Influence of Precursor Selection on Room-Temperature Ferromagnetism (RTFM)

The preparation of materials has always been optimized by selecting more suitable precursors. In 2019, Zn(NO_3_)_2_·6H_2_O and ZnCl_2_ were selected as zinc sources to prepare ZnO NPs [104]. The difference of *M_s_* was nearly four times, which may have been due to V_O_ in the samples produced by selecting different precursors.

### 3.2. Substrate Effects on Ferromagnetism

**Figure 12:**
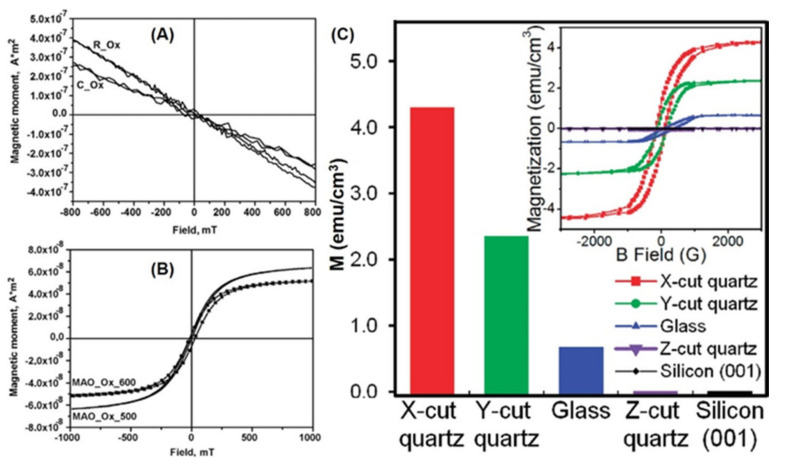
Substrate effects on RTFM. (**A**) M-H loops of ZnO TFs prepared by CVD on sapphire substrates (Reproduced with permission from [93]. Copyright 2019, Elsevier). (**B**) M-H loops of ZnO TFs deposited by CVD on (111) MgAl_2_O_4_ substrates (Reproduced with permission from [93]. Copyright 2019, Elsevier). (**C**) *M_s_* of ZnO TFs deposited on different substrates (Reproduced with permission from [106]. Copyright 2009, American Chemical Society).

Substrates have played an important role in material synthesis [4,93,94,105,106,107,108]. ZnO TFs have been grown on single-crystalline substrates by MOCVD [93]. M–H curves show that ZnO TFs on sapphire substrate are non-ferromagnetic (Figure 12A,B). However, the structural defect located on the substrate–film interface can cause RTFM in ZnO TFs. Interestingly, different substrates have been used to fabricate ZnO TFs with the same thickness by PED [94], and their ferromagnetic behaviors are very different. The ZnO TFs on silicon substrate show stronger ferromagnetism than the others, because more V_O_ can be created in the TFs [94]. The origin of RTFM may be V_O_.

ZnO TFs grown on sapphire substrates have been synthesized by PLD [105]. Only the samples on R-plane sapphire substrate exhibit RTFM. Furthermore, the results suggest that V_Zn_ is probably the reason for the RTFM. In addition, quartz, glass, and silicon have been selected as substrates to prepare ZnO TFs by PLD, and the RTFM in the samples may come from the coupling of unpaired electron spins induced by the oxygen 2p orbitals around the V_Zn_ and are enhanced by the in-plane compressive strain [106].

The modulation of RTFM by changing the substrate temperature has also been studied in ZnO TFs [107]. However, ZnO films grown at low substrate temperatures exhibit a larger *M_s_*. *M_s_* decreases with increasing the temperature. A similar result is found by Xu et al. [108]. Notably, the effect is different from that in TM-doped ZnO TFs [4].

### 3.3. Effect of Growth Conditions on RTFM

The RTFM can also be mediated by growth temperature. In 2012, Xu et al. [52] found that ZnO QDs with different sizes exhibited an obvious RTFM. Furthermore, the surface-volume ratio was closely related with the size-dependent ferromagnetism.

The size of QDs can also be controlled by milling time, which affects RTFM of QDs. Furthermore, it has been found that milling time is a superior way to mediate RTFM [49,57,58]. The grain size does not change monotonously with milling time, but *M_s_* will increase monotonously in a certain range. In fact, V_Zn_ can induce RTFM in ZnO QDs.

Similarly, the grain size can be changed by milling time, as reported by Kisan et al. [57]. Interestingly, the transition from PM to FM is realized by this strategy. UV-vis spectra [57] and positron annihilation spectra [109] show that V_O_ can affect the RTFM.

### 3.4. Influence of Post-Annealing on RTFM

Zhan et al. [110]. observed that RTFM can be produced by annealing in Ar. The magnetic field strength will affect the observed RTFM. When a magnetic field of 7T is applied, *M_s_* is as high as 2.7 emu/g. Furthermore, PL spectra indicate that RTFM originates from V_O_^+^.

Undoped ZnO TFs with RTFM have been obtained by PAD [89]. The magnetization of the sample can potentiate by annealing in O_2_ or H_2_ gas. Positron annihilation spectroscopy indicates that V_Zn_ can induce the RTFM. In addition, we have also fabricated ferromagnetic Zn_0.97_Co_0.03_O TFs by PAD [90]. The O_i_ and V_Zn_ can be mediated with the annealing. The PL spectra show that the RTFM is closely related to O_i_ and V_Zn_.

Changing annealing temperature is another strategy to mediate RTFM [53,54] The *H_c_*, *M_r_* and *M_s_* increase with decreasing the annealing temperature of the samples. PL spectra show that Zn_i_ and V_O_ related defects are closely related to the RTFM.

Interestingly, we have found that as-grown ZnO TFs can be transformed into centimeter-scale ZnO horizontal NWs arrays [43] by annealing in O_2_. With the extension of annealing time, the length becomes longer. After ZnO NWs arrays have been produced, the magnetic properties of the films transform PM into FM.

### 3.5. Dopping with Non-Magnetic Atoms

Notably, doping is also an effective way to modulate the RFTM of ZnO. Many research groups have attempted to look for compounds with RTFM since 2000, which can be obtained with doping transition metal elements into metal oxides such as Cr [4,111,112,113], Mn [2,3,4], Fe [114], Ni [115], Co [90,116,117,118], Sc [4,119,120], Ti [120], V [120], and Cu [120,121], and codoping such as CoFe [122], and MnCo [123]. However, experimental studies on TM-doped ZnO have produced inconsistent results and the mechanism of RTFM remains unclear. This promoted search for DMS based on alternative dopants. Manifestation of RTFM has also been undertaken by doping with non-magnetic dopants such as H [26,98,99,100,101,102,103,104,105,106,107,108,109,110,124,125], N [126,127], C [128,129,130,131], and S [47,78]. Interestingly, the observation of RTFM strongly suggests the presence of a spin-split band with a non-zero spin-orbit coupling in H-ZnO single crystals [100]. In addition, incorporation of N at 60° in ZnO induces the stronger FM, higher band gap reduction, larger reduction in surface roughness and higher reduction in the grains at RT [126]. Carbon-doped ZnO films show an intrinsic RTFM, which originates from the Zn-C system in the ZnO environment [128]. Meanwhile, thiol-capped nanotube arrays have exhibited a higher *M_s_* than the nanowire arrays due to its larger surface-to-volume ratio and thus higher density of Zn–S bond spins. The observed dependence of the magnetic anisotropy of ZnO NTs haves suggested that the change in the ordering of Zn–S spins on the crystal facets can affect the extent of magnetic ordering and alter the preferred magnetization direction [78].

### 3.6. Capping

Capping organic molecules is a common method to mediate RTFM of non-magnetic materials. In 2007, Garcia et al. [36] controlled the ferromagnetism by capping ZnO NPs with three different organic molecules. The band structure was mediated with capping molecules, and then the RTFM was induced from DM.

## 4. Conclusions and Outlook

The present literature analysis reveals that the amount of research focused on un-doped ZnO nanostructures with RFTM has been increasing over the past two decades. We have successfully prepared ferromagnetic nanostructures, including thin films, horizontal arrays and vertical arrays. RTFM could be readily modulated by non-doping means including precursor selection, substrate effects, growth conditions, post-annealing, and capping. Some novel ways to drive the transition from DM to FM in undoped ZnO nanostructures have delivered interesting results, as shown in Figure 12.

The size-dependent RTFM in ZnO QDs/NPs (Figure 3) can be observed [52]. *M_s_* is increased gradually by decreasing the diameters of ZnO QDs/NPs. However, in the nanowire system, the change of diameter has no obvious effect on RTFM (Figure 7). Interestingly, RTFM can be induced from non-magnetic ZnO nanosheets and TFs by reducing the thickness (Figure 10 and Figure 12). Notably, the magnetic anisotropy of ZnO nanostructures should be further studied.

Through theoretical calculation, it has been found that there is a driving force of phase transformation in ZnO. Therefore, fabrication of ultrathin (thickness < 2 nm) freestanding ZnO structures remains challenging. Amazingly, Taniguchi et al. [45] have reported that organic layers could allow the formation of ZnO nanosheets (~1.5 nm) with RTFM. In addition, Zhao et al. [132]. have presented the phase transitions of quantum-confined ZnO NWs by in-site TEM. Experimentally, it is still a great challenge to study RTFM in quantum-bound ZnO nanostructures.

Even though a variety of magnetic nanostructures have been prepared and studied, there are still many problems worthy of further study. (1) Development of new ferromagnetic nanostructures, such as nano-octagonal stars [133]. In the past, the morphology characterization mainly focused on 2D characterization. Recently, 3D electron tomography has emerged to analyze the 3D structure of nanostructures. In addition, the self-assembly of nanostructures and the evolution of ferromagnetism were further studied by an in situ method [133,134]. (2) Development of some emerging methods to prepare and study the nanostructure. (3) New characterization methods have been employed to investigate the nanostructures with RTFM [135,136]. The crystal nucleation process of FePt NPs in four dimensions [135] has been studied by atomic electron tomography (AET) [135]. In addition, electron tomography (ET) has revealed FeCo nano-octopods with RTFM. (4) The mechanism of undoped ZnO with RTFM needs further study.

## Figures and Tables

**Table 1:** Summary of research on ferromagnetic (FM) zinc oxide. RTFM (room-temperature ferromagnetism).

Topic	Year	References
RTFM of TM-doped ZnO films	2005	[23]
RTFM of TM-doped ZnO films	2008	[4]
Theoretical and experimental studies of ZnO-based DMS	2010	[24]
RTFM of capped ZnO nanoparticles	2011	[25]
Defect-induced magnetism in undoped ZnO	2013	[26]
RTFM in ZnO nanomaterials	2013	[27]
ZnO-from dilute magnetic doping to spin transport	2014	[28]
Defect-driven RTFM in undoped ZnO	2015	[6]
Surface and/or interface RTFM of undoped ZnO nanoparticles	2015	[29]
RTFM in ZnO: TM	2016	[30]
Vacancy defect-induced RTFM in undoped ZnO nanostructures	2017	[31]
ZnO-based dilute magnetic semiconductor	2020	[32]

## Data Availability

Not applicable.

## References

[1] Wolf S.A., Awschalom D.D., Buhrman R.A., Daughton J.M., von Molnár S., Roukes M.L., Chtchelkanova A.Y., Treger D.M. (2001). Spintronics: A Spin-Based Electronics Vision for the Future. Science.

[2] Sharma P., Gupta A., Rao K.V., Owens F.J., Sharma R., Ahuja R., Osorio-Guillén J., Johansson B., Gehring G.A. (2003). Ferromagnetism above room temperature in bulk and transparent thin films of Mn-doped ZnO. Nat. Mater..

[3] Kundaliya D.C., Ogale S.B., Lofland S., Dhar S., Metting C.J., Shinde S.R., Ma Z., Varughese B., Ramanujachary K., Salamanca-Riba L. (2004). On the origin of high-temperature ferromagnetism in the low-temperature-processed Mn–Zn–O system. Nat. Mater..

[4] Pan F., Song C., Liu X.J., Yang Y.C., Zeng F. (2008). Ferromagnetism and possible application in spintronics of transition-metal-doped ZnO films. Mat. Sci. Eng. R..

[5] Herng T.S., Qi D., Berlijn T., Yi J., Yang K., Dai Y., Feng Y.P., Santoso I., Sánchez-Hanke C., Gao X.Y. (2010). Room-Temperature Ferromagnetism of Cu-Doped ZnO Films Probed by Soft X-ray Magnetic Circular Dichroism. Phys. Rev. Lett..

[6] Ning S., Zhan P., Xie Q., Wang W., Zhang Z. (2015). Defects-Driven Ferromagnetism in Undoped Dilute Magnetic Oxides: A Review. J. Mater. Sci. Technol..

[7] Hong N.H., Sakai J., Prellier W., Hassini A., Ruyter A., Gervais F. (2004). Ferromagnetism in transition-metal-doped TiO_2_ thin films. Phys. Rev. B..

[8] Matsumoto Y., Murakami M., Shono T., Hasegawa T., Fukumura T., Kawasaki M., Ahmet P., Chikyow T., Koshihara S.-Y., Koinuma H. (2001). Room-Temperature Ferromagnetism in Transparent Transition Metal-Doped Titanium Dioxide. Science.

[9] Xu X.H., Blythe H.J., Ziese M., Behan A.J., Neal J.R., Mokhtari A., Ibrahim R.M., Fox A.M., Gehring G.A. (2006). Carrier-induced ferromagnetism in n-type ZnMnAlO and ZnCoAlO thin films at room temperature. New J. Phys..

[10] Yoon S.D., Chen Y.J., Yang A., Goodrich T.L., Zuo X., Arena D.A., Ziemer K., Vittoria C., Harriset V.G. (2006). Oxygen-defect-induced magnetism to 880 K in semiconducting anatase TiO_2−δ_ films. J. Phys.-Condens. Mat..

[11] Ogale S.B., Choudhary R.J., Buban J.P., Lofland S.E., Shinde S.R., Kale S.N., Kulkarni V.N., Higgins J., Lanci C., Simpson J.R. (2003). High temperature ferromagnetism with a giant magnetic moment in transparent Co-doped SnO_2_. Phys. Rev. Lett..

[12] Coey J.M.D., Douvalis A.P., Fitzgerald C.B., Venkatesan M. (2004). Ferromagnetism in Fe-doped SnO_2_ thin films. Appl. Phys. Lett..

[13] Gao Y., Hou Q.Y., Liu Y. (2019). Effect of Fe Doping and Point Defects (VO and VSn) on the Magnetic Properties of SnO_2_. J. Supercond. Nov. Magn..

[14] Lin L., Wang P., Huang J., Yu W., Tao H., Zhu L., Zhang Z. (2019). Investigation on Electronic Structures and Magnetic Properties of (Mn, Ga) Co-doped SnO_2_. J. Supercond. Nov. Magn..

[15] Zhang C., Zhou M., Zhang Y., Hao W., Sun L., Cao E., Yang Z. (2019). Effects of Oxygen Vacancy on the Magnetic Properties of Ni-Doped SnO_2_ Nanoparticles. J. Supercond. Nov. Magn..

[16] Pereira M.S., Mendes G.M.S.L., Ribeiro T.S., Silva M.R., Vasconcelos I.F. (2020). Influence of Thermal-Treatment Effects on the Structural and Magnetic Properties of Sn1−xFexO_2_ Nanopowders Produced by Mechanical Milling. J. Supercond. Nov. Magn..

[17] He J., Xu S.F., Yoo Y.K., Xue Q.Z., Lee H.C., Cheng S.F., Xiang X.D., Dionne G.F., Takeuchiet I. (2005). Room temperature ferromagnetic n-type semiconductor in (In_1−x_Fe_x_)_2_O_3−σ_. Appl. Phys. Lett..

[18] Jiang F.-X., Xu X.-H., Zhang J., Fan X.-C., Wu H.-S., Gehring G.A. (2010). Role of carrier and spin in tuning ferromagnetism in Mn and Cr-doped In_2_O_3_ thin films. Appl. Phys. Lett..

[19] Venkatesan M., Fitzgerald C.B., Coey J.M.D. (2004). Unexpected magnetism in a dielectric oxide. Nature.

[20] Coey J.M.D., Venkatesan M., Stamenov P., Fitzgerald C.B., Dorneles L.S. (2005). Magnetism in hafniumk dioxide. Phys. Rev. B.

[21] Hong N.H., Poirot N., Sakai J. (2006). Evidence for magnetism due to oxygen vacancies in Fe-doped HfO_2_ thin films. Appl. Phys. Lett..

[22] Dietl T., Ohno H., Matsukura F., Cibert J., Ferrand D. (2000). Zener model description of ferromagnetism in zinc-blende magnetic semico,,nductors. Science.

[23] Coey J.M.D. (2005). d^0^ ferromagnetism. Solid State Sci..

[24] Avrutin V., Izyumskaya N., Üzgür O., Silversmith D.J., Morkoç H. (2010). Ferromagnetism in ZnO- and GaN-based diluted magnetic Semiconductors: Achievements and challenges. Proc. IEEE.

[25] Hernando A., Crespo P., Garcia M.A., Coey J.M.D., Ayuela A., Echenique P.M. (2011). Revisiting magnetism of capped Au and ZnO nanoparticles: Surface band structure and atomic orbital with giant magnetic moment. Phys. Status Solidi B.

[26] Esquinazi P., Hergert W., Spemann D., Setzer A., Ernst A. (2013). Defect-Induced Magnetism in Solids. IEEE Trans. Magn..

[27] Singh R. (2013). Unexpected magnetism in nanomaterials. J. Magn. Magn. Mater..

[28] Opel M., Goennenwein S.T.B., Althammer M., Nielsen K.-W., Karrer-Müller E.-M., Bauer S., Senn K., Schwark C., Weier C., Güntherodt G. (2014). Zinc oxide -From dilute magnetic doping to spin transport. Phys. Status Solidi B.

[29] Zhang J., Yu L., Song Q., Du Y. (2015). Tunable surface and/or interface ferromagnetism of ZnO nanoparticles. Ann. Phys..

[30] Semisalova A.S., Orlov A., Smekhova A., Gan’shina E., Perov N., Anwand W., Potzger K., Lähderanta E., Granovsky A., Zhukov A. (2016). Above room temperature ferromagnetism in dilute magnetic oxide semiconductors. Novel Functional Magnetic Materials: Fundamentals and Applications.

[31] Qi B., Ólafsson S., Gíslason H. (2017). Vacancy defect-induced d0 ferromagnetism in undoped ZnO nanostructures: Controversial origin and challenges. Prog. Mater. Sci..

[32] Aravind A., Jayaraj M.K., Jayaraj M.K. (2020). ZnO-based dilute magnetic semiconductors. Nanostructured Metal Oxides and Devices: Optical and Electrical Properties.

[33] Li X.-L., Xu X.-H. (2019). Homogeneous and inhomogeneous magnetic oxide semiconductors. Chin. Phys. B.

[34] Galland D., Herve A. (1970). ESR spectra of the zinc vacancy in ZnO. Phys. Lett. A.

[35] Potzger K., Zhou S.Q., Grenzer J., Helm M., Fassbender J. (2008). An easy mechanical way to create ferromagnetic defective ZnO. Appl. Phys. Lett..

[36] Garcia M.A., Merino J.M., Fernández P.E., Quesada A., De la Venta J., Ruíz G.M.L., Hernando A. (2007). Magnetic properties of ZnO nanoparticles. Nano Lett..

[37] Köseoğlu Y. (2014). A simple microwave-assisted combustion synthesis and structural, optical and magnetic characterization of ZnO nanoplatelets. Ceram. Int..

[38] Sundaresan A., Bhargavi R., Rangarajan N., Siddesh U., Rao C.N.R. (2006). Ferromagnetism as a universal feature of nanoparticles of the otherwise nonmagnetic oxides. Phys. Rev. B.

[39] Banerjee S., Mandal M., Gayathri N., Sardar M. (2007). Enhancement of ferromagnetism upon thermal annealing in pure ZnO. Appl. Phys. Lett..

[40] Hong N.H., Sakai J., Brizé V. (2007). Observation of ferromagnetism at room temperature in ZnO thin films. J. Phy. Condens. Matter.

[41] Yin X., Wang Y.Z., Jacobs R., Shi Y.Q., Szlufarska I., Morgan D., Wang X.D. (2019). Massive vacancy concentration yields strong room-temperature ferromagnetism in two-dimensional ZnO. Nano Lett..

[42] Ren H., Liu Y., Zhang L., Liu K. (2019). Synthesis, properties, and applications of large-scale two-dimensional materials by polymer-assisted deposition. J. Semicond..

[43] Ren H., Xiang G., Luo J., Yang D., Zhang X. (2018). Direct catalyst-free self-assembly of large area of horizontal ferromagnetic ZnO nanowire arrays. Mater. Lett..

[44] Luo J., Ren H., Zhang X., Xiang G. (2020). Fabrication of vertically aligned ferromagnetic ZnO nanopillar arrays on sapphire substrates by polymer-assisted deposition. AIP Adv..

[45] Taniguchi T., Yamaguchi K., Shigeta A., Matsuda Y., Hayami S., Shimizu T., Matsui T., Yamazaki T., Funatstu A., Makinose Y. (2013). Enhanced and Engineered d0Ferromagnetism in Molecularly-Thin Zinc Oxide Nanosheets. Adv. Funct. Mater..

[46] Xu Q., Zhou S., Schmidt H. (2009). Magnetic properties of ZnO nanopowders. J. Alloys Compd..

[47] Chaboy J., Boada R., Piquer C., Marco M.A.L., García-Hernández M., Carmona N., Llopis J., Gonzalez M.L.R., Gonzalez-Calbet J.M., Fernandez J. (2010). Evidence of intrinsic magnetism in capped ZnO nanoparticles. Phys. Rev. B.

[48] Xu Q., Wen Z., Zhang H., Qi X., Zhong W., Xu L., Wu D., Shen K., Xu M. (2011). Room temperature ferromagnetism in ZnO prepared by microemulsion. AIP Adv..

[49] Phan T.-L., Zhang Y.D., Yang D.S., Nghia N.X., Thanh T.D., Yu S.C. (2013). Defect-induced ferromagnetism in ZnO nanoparticles prepared by mechanical milling. Appl. Phys. Lett..

[50] Lemine O.M. (2016). Induced Room-Temperature Ferromagnetism in Un-doped Nanocrystalline Metal Oxide Powders Obtained by Mechanical Milling: A Review. J. Supercond. Nov. Magn..

[51] Zhao C., Huang Y., Abiade J.T. (2012). Ferromagnetic ZnO nanoparticles prepared by pulsed laser deposition in liquid. Mater. Lett..

[52] Xu X., Xu C., Dai J., Hu J., Li F., Zhang S. (2012). Size Dependence of Defect-Induced Room Temperature Ferromagnetism in Undoped ZnO Nanoparticles. J. Phys. Chem. C.

[53] Zhang Y., Xie E. (2010). Nature of room-temperature ferromagnetism from undoped ZnO nanoparticles. Appl. Phys. A.

[54] Naji Aljawfi R., Rahman F., Shukla D.K. (2013). Effect of the annealing temperature on the structural and magnetic properties of ZnO nanoparticles. Mater. Lett..

[55] Ghose S., Gogurla N., Ranganathan R., Jana D. (2016). The simultaneous emergence of free exciton emission and d0 ferromagnetism for undoped ZnO nanoparticles. RSC Adv..

[56] Ghose S., Rakshit T., Ranganathan R., Jana D. (2015). Role of Zn-interstitial defect states on d0 ferromagnetism of mechanically milled ZnO nanoparticles. RSC Adv..

[57] Kisan B., Alagarsamy P. (2014). Room temperature ferromagnetism in finite sized ZnO nanoparticles. Phys. B Condens. Matter.

[58] Kisan B., Kumar J., Alagarsamy P. (2020). Experimental and first-principles study of defect-induced electronic and magnetic properties of ZnO nanocrystals. J. Phys. Chem. Solids.

[59] Wang D., Chen Z.Q., Wang D.D., Qi N., Gong J., Cao C.Y., Tang Z. (2010). Positron annihilation study of the interfacial defects in ZnO nanocrystals: Correlation with ferromagnetism. J. Appl. Phys..

[60] Ghose S., Sarkar A., Chattopadhyay S., Chakrabarti M., Das D., Rakshit T., Ray S.K., Jana D. (2013). Surface defects induced ferromagnetism in mechanically milled nanocrystalline ZnO. J. Appl. Phys..

[61] Xue X., Liu L., Wang Z., Wu Y. (2014). Room-temperature ferromagnetism in hydrogenated ZnO nanoparticles. J. Appl. Phys..

[62] Gao D., Zhang Z., Fu J., Xu Y., Qi J., Xue D. (2009). Room temperature ferromagnetism of pure ZnO nanoparticles. J. Appl. Phys..

[63] Pazhanivelu V., Blessington Selvadurai A.P., Murugaraj R. (2015). Unexpected ferromagnetism in Ist group elements doped ZnO based DMS nanoparticles. Mater. Lett..

[64] Wangensteen T., Dhakal T., Merlak M., Mukherjee P., Phan M., Chandra S., Srikanth H., Witanachchi S. (2011). Growth of uniform ZnO nanoparticles by a microwave plasma process. J. Alloys Compd..

[65] Yan Z., Ma Y., Wang D., Wang J., Gao Z., Song T. (2008). Surfactant-Free Fabrication of ZnO Spheres and Pseudospherical Structures. J. Phys. Chem. C.

[66] Yi J.B., Pan H., Lin J.Y., Ding J., Feng Y.P., Thongmee S., Liu T., Gong H., Wang L. (2008). Ferromagnetism in ZnO Nanowires Derived from Electro-deposition on AAO Template and Subsequent Oxidation. Adv. Mater..

[67] Yan Z., Ma Y., Wang D., Wang J., Gao Z., Wang L., Yu P., Song T. (2008). Impact of annealing on morphology and ferromagnetism of ZnO nanorods. Appl. Phys. Lett..

[68] Kumar S., Kim Y., Koo B., Gautam S., Chae K.H., Kumar R., Lee C. (2009). Room temperature ferromagnetism in chemically synthesized ZnO rods. Mater. Lett..

[69] Podila R., Queen W., Nath A., Arantes J.T., Schoenhalz A.L., Fazzio A., Dalpian G.M., He J., Hwu S.J., Skove M.J. (2010). Origin of FM Ordering in Pristine Micro- and Nanostructured ZnO. Nano Lett..

[70] Panigrahy B., Aslam M., Misra D.S., Ghosh M., Bahadur D. (2010). Defect-Related Emissions and Magnetization Properties of ZnO Nanorods. Adv. Funct. Mater..

[71] Xing G.Z., Wang D.D., Yi J.B., Yang L.L., Gao M., He M., Yang J.H., Ding J., Sum T.C., Wu T. (2010). Correlated d^0^ ferromagnetism and photoluminescence in undoped ZnO nanowires. Appl. Phys. Lett..

[72] Jana A., Sujatha Devi P., Mitra A., Bandyopadhyay N.R. (2013). Synthesis of blue emitting ZnO nanorods exhibiting room temperature ferromagnetism. Mater. Chem. Phys..

[73] Ghosh B., Ray S.C., Pontsho M., Sarma S., Mishra D.K., Wang Y.F., Pong W.F., Strydom A.M. (2018). Defect induced room temperature ferromagnetism in single crystal, poly-crystal, and nanorod ZnO: A comparative study. J. Appl. Phys..

[74] Xu X.Y., Xu C.X., Lin Y., Ding T., Fang S.J., Shi Z.L., Xia W.W., Hu J.G. (2012). Surface photoluminescence and magnetism in hydrothermally grown undoped ZnO nanorod arrays. Appl. Phys. Lett..

[75] Li Z., Zhong W., Li X., Zeng H., Wang G., Wang W., Yang Z., Zhang Y. (2013). Strong room-temperature ferromagnetism of pure ZnO nanostructure arrays via colloidal template. J. Mater. Chem. C.

[76] Xu X., Xu C., Lin Y., Li J., Hu J. (2013). Comparison on Photoluminescence and Magnetism between Two Kinds of Undoped ZnO Nanorods. J. Phys. Chem. C.

[77] Singh S.B., Wang Y.-F., Shao Y.-C., Lai H.-Y., Hsieh S.-H., Limaye M.V., Chuang C.-H., Hsueh H.-C., Wang H., Chiou J.-W. (2014). Observation of the origin of d0magnetism in ZnO nanostructures using X-ray-based microscopic and spectroscopic techniques. Nanoscale.

[78] Deng S.Z., Fan H.M., Wang M., Zheng M.R., Yi J.B., Wu R.Q., Tan H.R., Sow C.H., Ding J., Loh Y.P.K.P. (2010). Thiol-capped ZnO nanowire/nanotube arrays with tunable magnetic properties at toom temperature. ACS Nano.

[79] Bie X., Wang C., Ehrenberg H., Wei Y., Chen G., Meng X., Zou G., Du F. (2010). Room-temperature ferromagnetism in pure ZnO nanoflowers. Solid State Sci..

[80] Gupta J., Bhargava P., Bahadur D. (2014). Morphology dependent photocatalytic and magnetic properties of ZnO nanostructures. Phys. B Condens. Matter.

[81] Motaung D.E., Mhlongo G.H., Nkosi S.S., Malgas G.F., Mwakikunga B.W., Coetsee E., Swart H.C., Abdallah H.M.I., Moyo T., Ray S.S. (2014). Shape-Selective Dependence of Room Temperature Ferromagnetism Induced by Hierarchical ZnO Nanostructures. ACS Appl. Mater. Interfaces.

[82] Hong J.-I., Choi J., Jang S.S., Gu J., Chang Y., Wortman G., Snyder R.L., Wang Z.L. (2012). Magnetism in Dopant-Free ZnO Nanoplates. Nano Lett..

[83] Yang H.-F., Tang L.-Z., Sun Q., Sun L., Li Z.-H., Ren S.-X. (2018). Ferromagnetism in High-Surface-Area ZnO Nanosheets Prepared by a Template-Assisted Hydrothermal Method. Chin. Phys. Lett..

[84] Wang Q., Sun Q., Chen G., Kawazoe Y., Jena P. (2008). Vacancy-induced magnetism in ZnO thin films and nanowires. Phys. Rev. B.

[85] Kapilashrami M., Xu J., Ström V., Rao K.V., Belova L. (2009). Transition from ferromagnetism to diamagnetism in undoped ZnO thin films. Appl. Phys. Lett..

[86] Straumal B., Mazilkin A., Protasova S., Myatiev A., Goering E., Baretzky B. (2011). Amorphous grain boundary layers in the ferromagnetic nanograined ZnO films. Thin Solid Films.

[87] Tietze T., Audehm P., Chen Y.C., Schütz G., Straumal B.B., Protasova S.G., Mazilkin A.A., Straumal P.B., Prokscha T., Luetkens H. (2015). Interfacial dominated ferromagnetism in nanograined ZnO: A μSR and DFT study. Sci. Rep..

[88] Xing G.Z., Lu Y.H., Tian Y.F., Yi J.B., Lim C.C., Li Y.F., Li G.P., Wang D.D., Yao B., Ding J. (2011). Defect-induced magnetism in undoped wide band gap oxides: Zinc vacancies in ZnO as an example. AIP Adv..

[89] Ren H.T., Xiang G., Gu G., Wang W.J., Zhang P., Wang B.Y., Cao X.Z. (2012). Zinc Vacancy-induced room-temperature ferromagnetism in undoped ZnO thin films. J. Nanomat..

[90] Ren H.T., Xiang G., Gu G.X., Zhang X. (2014). Enhancement of ferromagnetism of ZnO:Co nanocrystals by post-annealing treatment: The role of oxygen interstitials and zinc vacancies. Mater. Lett..

[91] Mal S., Narayan J., Nori S., Prater J., Kumar D. (2010). Defect-mediated room temperature ferromagnetism in zinc oxide. Solid State Commun..

[92] Kapilashrami M., Xu J., Biswas A., Tamaki T., Sharma P., Rao K., Belova L. (2010). Coexistence of ultraviolet photo-response and room-temperature ferromagnetism in polycrystalline ZnO thin films. Mater. Lett..

[93] Burova L.I., Perov N.S., Semisalova A.S., Kulbachinskii V.A., Vladimir G., Kytin V.G., Roddatis V.V., Alexander L., Vasiliev A.L., Andrey R. (2012). Effect of the nanostructure on room temperature ferromagnetism and resistivity of undoped ZnO thin films grown by chemical vapor deposition. Thin Solid Films.

[94] Zhan P., Wang W., Xie Z., Li Z., Zhang Z., Zhang P., Wang B., Cao X. (2012). Substrate effect on the room-temperature ferromagnetism in un-doped ZnO films. Appl. Phys. Lett..

[95] Zhao J.G., Zhang W.Y., An X.Y., Liu Z.J., Xie E.Q., Yang C., Chen L.L. (2014). Room-temperature ferromagnetism in ZnO nanoparticles by electrospinning. Nanosci. Nanotech. Lett..

[96] Zhang X., Zhang W., Zhang X., Xu X., Meng F., Tang C.C. (2014). Defects Induced Room Temperature Ferromagnetism in ZnO Thin Films. Adv. Condens. Matter Phys..

[97] Mishra D., Kumar P., Sharma M.K., Das J., Singh S., Roul B., Varma S., Chatterjee R., Srinivasu V., Kanjilal D. (2010). Ferromagnetism in ZnO single crystal. Phys. B Condens. Matter.

[98] Sanchez N., Gallego S., Cerdá J., Muñoz M.C. (2010). Tuning surface metallicity and ferromagnetism by hydrogen adsorption at the polar ZnO(0001) surface. Phys. Rev. B.

[99] Khalid M., Esquinazi P., Spemann D., Anwand W., Bräuer G. (2011). Hydrogen-mediated ferromagnetism in ZnO single crystals. New J. Phys..

[100] Khalid M., Esquinazi P.D. (2012). Hydrogen-induced ferromagnetism in ZnO single crystals investigated by magnetotransport. Phys. Rev. B.

[101] Khazanov E.N., Taranov A.V., Salamatov E.I., Shevchenko E.V., Charnaya E.V. (2017). Features of defects of the crystal structure and magnetic properties of an undoped ZnO monocrystal. J. Commun. Technol. Electron..

[102] Xia Z.B., Sha J., Fang Y.J., Wan Y.T., Wang Z.L., Wang Y.W. (2010). Purposed built ZnO/Zn_5_(OH)_8_Ac_2_·2H_2_O architectures by hydrothermal synthesis. Cryst. Growth Des..

[103] Xia Z.B., Wang Y.W., Fang Y.J., Wan Y.T., Xia W., Sha J. (2011). Understanding the origin of ferromagnetism in ZnO porous microspheres by systematic investigations of the thermal decomposition of Zn_5_(OH)_8_Ac_2_·2H_2_O to ZnO. J. Phys. Chem. C.

[104] Tóthová E., Senna M., Yermakov A., Kováč J., Dutková E., Hegedüs M., Kaňuchová M., Baláž M., Bujňáková Z.L., Briančin J. (2019). Zn source-dependent magnetic properties of undoped ZnO nanoparticles from mechanochemically derived hydrozincite. J. Alloys Compd..

[105] Khalid M., Ziese M., Setzer A.P., Esquinazi P.M., Lorenz M.H., Hochmuth H., Grundmann M., Spemann D., Butz T., Brauer G. (2009). Defect-induced magnetic order in pure ZnO films. Phys. Rev. B.

[106] Ong C.S., Herng T.S., Huang X.L., Feng Y.P., Ding J. (2011). Strain-Induced ZnO Spinterfaces. J. Phys. Chem. C.

[107] Mal S., Nori S., Narayan J., Prater J.T. (2011). Defect-mediated ferromagnetism and controlled switching characteristics in ZnO. J. Mater. Res..

[108] Xu Q., Wen Z., Xu L., Gao J., Wu D., Shen K., Qiu T., Tang S., Xu M. (2011). Room temperature ferromagnetic pure ZnO. Phys. B Condens. Matter.

[109] Sanyal D., Chakrabarti M., Roy T.K., Chakrabarti A. (2007). The origin of ferromagnetism and defect-magnetization correlation in nanocrystalline ZnO. Phys. Lett. A.

[110] Zhan P., Wang W., Xie Q., Li Z., Zhang Z. (2012). Enhanced room-temperature ferromagnetism in un-doped ZnO thin films by thermal annealing in a strong magnetic field. J. Appl. Phys..

[111] Hong N.H., Sakai J., Huong N.T., Poirot N., Ruyter A. (2005). Role of defects in tuning ferromagnetism in diluted magnetic oxide thin films. Phys. Rev. B.

[112] Liu H., Zhang X., Li L., Wang Y.X., Gao K.H., Li Z.Q., Zheng R., Ringer S., Zhang B. (2007). Role of point defects in room-temperature ferromagnetism of Cr-doped ZnO. Appl. Phys. Lett..

[113] Wang Q., Sun Q., Jena P., Kawazoe Y. (2005). Magnetic coupling between Cr atoms doped at bulk and surface sites of ZnO. Appl. Phys. Lett..

[114] Thurber A.P., Beausoleil II G.L., Alanko G.A., Anghel J.J., Jones M.S., Johnson L.M., Zhang J.H., Hanna C.B., Tenne D.A., Punnoose A. (2011). Magnetism of ZnO nanoparticles: Dependence on crystallite size and surfactant coating. J. Appl. Phys..

[115] Rana A.K., Kumar Y., Rajput P., Jha S.N., Bhattacharyya D., Shirage P.M. (2017). Search for origin of room temperature ferromagnetism properties in Ni-doped ZnO nanostructure. ACS Appl. Mater. Inter..

[116] Wojnarowicz J., Kusnieruk S., Chudoba T., Gierlotka S., Lojkowski W., Knoff W., Lukasiewicz M.I., Witkowski B., Wolska A., Klepka M. (2015). Paramagnetism of cobalt-doped ZnO nanoparticles obtained by microwave solvothermal synthesis. Beilstein J. Nanotechnol..

[117] MacManus-Driscoll J.L., Khare N., Liu Y., Vickers M.E. (2007). Structural Evidence for Zn Intersititials in Ferromagnetic Zn1–xCoxO Films. Adv. Mater..

[118] Chen R., Luo F.C., Liu Y.Z., Song Y., Dong Y., Wu S., Cao J.H., Yang F.Y., N’Diaye A., Shafer P. (2021). Tunable room-temperature ferromagnetism in Co-doped two-dimensional van der Waals ZnO. Nat. Commun..

[119] Coey J., Venkatesan M., Fitzgerald C., Dorneles L., Stamenov P., Lunney J. (2004). Anisotropy of the magnetization of a dilute magnetic oxide. J. Magn. Magn. Mater..

[120] Venkatesan M., Fitzgerald C.B., Lunney J.G., Coey J.M.D. (2004). Anisotropic Ferromagnetism in Substituted Zinc Oxide. Phys. Rev. Lett..

[121] Buchholz D.B., Chang R.P.H., Song J.H., Ketterson J.B. (2005). Room-temperature Ferromagnetism in Cu-doped ZnO Thin Films. Appl. Phys. Lett..

[122] Huang J.C.A., Hsu H.S. (2005). Inspection of magnetic semiconductor and clustering structure in CoFe-doped ZnO films by bias-dependent impedance spectroscopy. Appl. Phys. Lett..

[123] Du C.L., Gu Z.B., Lu M.H., Wang J., Zhang S.T., Zhao J., Cheng G.X., Heng H., Chen Y.F. (2006). Raman spectroscopy of (Mn, Co)-codoped ZnO films. J. Appl. Phys..

[124] Robaina O.V., Cabrera A.F., Meyer M., Romano R.M., Cruz A.F., Torres C.E.R. (2019). Room-Temperature Ferromagnetism Induced by High-Pressure Hydrogenation of ZnO. J. Phys. Chem. C.

[125] Salimian A., Abul H.A., Aminishahsavarani A., Upadhyaya H. (2021). Hypothesis on the influence of the magnetic behaviour of hydrogen doped Zinc oxide during its plasma sputtering process. Coatings.

[126] Hariwal R.V., Malik H.K., Negi A., Kandasami A. (2018). Controlling room temperature ferromagnetism and band gap in ZnO nanostructured thin films by varying angle of implantation. RSC Adv..

[127] Jindal K., Tomar M., Katiyar R., Gupta V. (2015). Transition from diamagnetic to ferromagnetic state in laser ablated nitrogen doped ZnO thin films. AIP Adv..

[128] Pan H., Yi J.B., Shen L., Wu R.Q., Yang J.H., Lin J.Y., Feng Y.P., Ding J., Van L.H., Yin J.H. (2007). Room-temperature ferromagnetism in Carbon-doped ZnO. Phys. Rev. Lett..

[129] Nagare B.J., Chacko S., Kanhere D.G. (2010). Ferromagnetism in Carbon-Doped Zinc Oxide Systems. J. Phys. Chem. A.

[130] Pham A., Assadi M.H.N., Zhang Y.B., Yu A.B., Li S. (2011). Weak d^0^ magnetism in C and N doped ZnO. J. Appl. Phys..

[131] Kumar P., Malik H.K., Asokan K., Kandasami A. (2015). Tuning of optical bandgap and magnetization of C-implanted ZnO thin films. EPL Europhys. Lett..

[132] Zhao P., Guan X., Zheng H., Jia S., Li L., Liu H., Zhao L., Sheng H., Meng W., Zhuang Y. (2019). Surface- and Strain-Mediated Reversible Phase Transformation in Quantum-Confined ZnO Nanowires. Phys. Rev. Lett..

[133] Garnero C., Pierrot A., Gatel C., Marcelot C., Arenal R., Florea I., Mantel A.B., Soulantica K., Poveda P., Chaudret B. (2021). Single-crystalline body centered FeCo nano-octopods: From one-pot chemical growth to a complex 3D magnetic configuration. Nano Lett..

[134] Ye S., Chen Z., Ha Y.-C., Wiley B.J. (2014). Real-Time Visualization of Diffusion-Controlled Nanowire Growth in Solution. Nano Lett..

[135] Zhou J.H., Yang Y.S., Yang Y., Kim D.S., Yuan A., Tian X.Z., Ophus C., Sun F., Schmid A.K., Nathanson M. (2019). Observing crystal nucleation in four dimensions using atomic electron tomography. Nature.

[136] Schreiber R.E., Houben L., Wolf S.G., Leitus G., Lang Z.-L., Carbó J.J., Poblet Z.-L.L.J.J.C.J.M., Neumann R.E.S.R. (2016). Real-time molecular scale observation of crystal formation. Nat. Chem..

